# β-Catenin-Gli1 interaction regulates proliferation and tumor growth in medulloblastoma

**DOI:** 10.1186/s12943-015-0294-4

**Published:** 2015-02-03

**Authors:** Jenny Zinke, Fabian T Schneider, Patrick N Harter, Sonja Thom, Nicole Ziegler, Rune Toftgård, Karl H Plate, Stefan Liebner

**Affiliations:** Institute of Neurology (Edinger-Institute), Johann Wolfgang Goethe-University Frankfurt, Medical School, Heinrich-Hoffmann-Straße 7, 60528 Frankfurt, Germany; Center for Biosciences and Department of Biosciences and Nutrition, Karolinska Institutet, Novum, Huddinge, Sweden; German Cancer Consortium (DKTK) and German Cancer Research Center (DKFZ), Heidelberg, Germany; Current address: Department of Neuropathology, Institute of Pathology and Pathological Anatomy, Technical University Munich, Trogerstrasse 18, 81675 Munich, Germany

**Keywords:** β-catenin, Gli1, Interaction, Medulloblastoma, Senescence, p21, LiCl, GSK-3β

## Abstract

**Background:**

The Wnt/beta-catenin and the Hedgehog (Hh) pathway interact in various cell types while eliciting opposing or synergistic cellular effects. Both pathways are known as exclusive drivers of two distinct molecular subtypes of medulloblastoma (MB).

In sonic hedgehog (Shh)-driven MB, activation of Wnt signaling has been shown to suppress tumor growth by either beta-catenin-dependent or -independent inhibition of Shh signaling. However, mechanistic insight in how beta-catenin inhibits the Hh pathway is not known.

**Findings:**

Here we show that beta-catenin stabilization by the glycogen synthase kinase 3 inhibitor lithium chloride (LiCl) reduced growth of primary hedgehog-driven MB tumor spheres from patched heterozygous mice (Ptch^+/-^) *in vitro*. LiCl treatment of MB spheres down-regulated the Hh target Gli1, whereas the repressive Gli3 protein (Gli3R) was increased. Mechanistically, we show by co-immunoprecipitation and proximity ligation assay that stabilized beta-catenin physically interacts with Gli1, leading to Gli1 sequestration and inhibition of its transcriptional activity. Reduction of Hh signaling upon LiCl stimulation resulted in reduced proliferation, sphere self renewal, a G2/M arrest and induction of a senescent-like state, indicated by p21 upregulation and by increased staining of senescence-associated beta-galactosidase (SA-betaGal). Moreover, LiCl treatment of subcutaneously transplanted MB cells significantly reduced tumor initiation defined as “tumor take”. Although tumor progression was similar, LiCl-treated tumors showed decreased mitotic figures and phospho-histone H3 staining.

**Conclusion:**

We propose that beta-catenin stabilization increases its physical interaction with Gli1, leading to Gli1 degradation and inhibition of Hh signaling, thereby promoting tumor cell senescence and suppression of “tumor take” in mice.

**Electronic supplementary material:**

The online version of this article (doi:10.1186/s12943-015-0294-4) contains supplementary material, which is available to authorized users.

## Introduction

In medulloblastoma (MB), the most common malignant pediatric brain tumor, Wnt pathway-driven tumors represent one of four distinct molecular subgroups with particularly favorable prognosis [1-4]. MB of the sonic hedgehog (Shh) subgroup account for up to 25-30% of human MBs and carry frequently mutations in the Shh receptor *patched 1* (*ptch1)* [2,5-7]. The prognosis of Hh-driven MBs is less favorable then the one of Wnt-driven MB and despite current multimodality treatment, MB patients suffer from considerable treatment-induced side effects [8,9].

In the “canonical” Wnt pathway β-catenin acts as a transcription factor with members of the lymphoid enhancer factor (Lef)/T-cell factor family [10]. On Wnt ligand-mediated activation of a complex formed by frizzled receptors and low-density lipoprotein receptor-related protein 5/6, proteasomal degradation of β-catenin is inhibited by inactivating the destruction complex formed by glycogen synthase kinase 3 β (GSK-3β), adenomatous poliposis coli (APC), and axin. Hedgehog, and specifically Shh, functions as a mitogen driving proliferation of granule neuron precursors in the cerebellum [11]. In the absence of Shh, patched (Ptch), a 12-transmembrane spanning protein, represses smoothened (SMO) thereby inhibiting Hh signaling. SMO is a member of the 7-transmembrane spanning G-protein-coupled receptor-like superfamily. On Shh binding to Ptch its repressive function on SMO is released, thereby activating Gli1/2 dependent transcription. In the repressive state Gli1/2 proteins are phosphorylated, ubiquitinated and degraded [12].

Interestingly, Wnt and Shh were shown to interact in various cell types and organs during development and in the adult, while eliciting opposing or synergistic cellular effects. Generally, it has previously been shown that Shh inhibits the Wnt pathway during tongue papilla development and in squamous cell carcinoma [1,3,4,13,14].

Recently, also in MB interaction of the Wnt and Hh pathway has been shown, describing either a β-catenin-dependent or -independent inhibition of Shh signaling by Wnt [5,6,15].

However, neither mechanistic insight into β-catenin-mediated inhibition of the Hh pathway, nor the therapeutic potential of Wnt/β-catenin-activating drugs has been examined specifically in MB.

Here we show that the FDA-approved drug LiCl, which results in β-catenin stabilization via GSK-3 inhibition, suppressed formation of Ptch^+/-^ MB tumor spheres as well as tumor take in mice. We provide mechanistic evidence that this effect is dependent on the physical interaction of Gli1 and β-catenin, leading to Gli1 degradation, G2/M cell cycle arrest and cellular senescence.

## Findings

**Figure 1:**
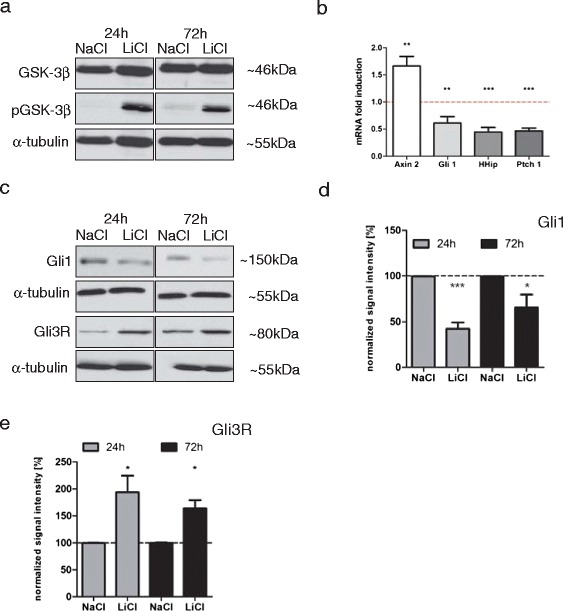
**Activation of Wnt/β-catenin signaling led to down-regulation of Hh target genes in primary Ptch** ^**+/-**^ **MB cells. (a)** Primary Ptch^+/-^ MB spheres treated for 24 h or 72 h with 10 mM NaCl or LiCl showed phosphorylated GSK-3β after treatment with LiCl. Whole cell lysates were probed with antibodies against GSK-3β, pGSK-3β (both Cell Signaling Technology, Danvers, MA, USA) and α-tubulin (Sigma-Aldrich, St. Louis, MO, USA) as a loading control. **(b)** Expression of Axin2, Gli1, Ptch1 and Hhip mRNA was evaluated in primary Ptch^+/-^ MB tumor cells after 72 h treatment with 10 mM NaCl or LiCl. Bars represent mean ± s.d. (*n* = 7, p-values left to right: **0.0064, *0.0186, ***˂0.0001, ***0.0002). Expression of Hh target genes was down-regulated by LiCl-treatment. **(c)** Gli1 protein levels decreased and Gli3R protein levels increased after LiCl treatment. Membranes were probed with antibodies against Gli1 (R&D Systems, Minneapolis, MN, USA) and α-tubulin (Sigma-Aldrich). **(d)** Quantification of signal intensity for Gli1 normalized to α-tubulin (ImageJ 1.47v software). Bars represent signal intensity after 24 h (grey) and 72 h (black) of NaCl and LiCl treatment. (*n* = 5, p-values left to right: *** < 0.0001, *0.0383). Gli1 levels decreased after treatment with LiCl. **(e)** Quantification of signal intensity for Gli3R normalized to α-tubulin (ImageJ 1.47v software). Bars represent signal intensity after 24 h (grey) and 72 h (black) of NaCl and LiCl treatment. (*n* = 3, p-values left to right: * < 0.0401, *0.0137). Gli3R levels increased after treatment with LiCl. (Analysis done in GraphPad Prism 5.01 software).

Primary MB cells isolated from Ptch^+/-^ mice [8] were treated with 10 mM LiCl *in vitro*. Inhibition of GSK-3 by LiCl, evidenced by GSK-3 phosphorylation (Figure 1a), led to reduced tumor sphere self-renewal (Additional file 1: Figure S1a) as well as to reduced tumor cell growth (Additional file 1: Figure S1b). In accordance, β-catenin stabilization and transcriptional activation, confirmed by increased Axin2 mRNA (Figure 1b) and protein levels (Additional file 2: Figure S2), led to the down-regulation of the Hh targets Gli1, Ptch1 and Hhip, on the mRNA level evidenced by qRT-PCR (see Table 1 for primer list), suggesting an inhibitory effect of β-catenin on Hh-induced transcription (Figure 1b). The decrease of Gli1 as well as an increase in β-catenin protein stability was verified by treatment with (2’Z,3’E)-6-Bromoindirubin-3’-oxime (6BIO), a non-FDA-approved but highly specific inhibitor of GSK-3 (Additional file 3: Figure S3) [16].

**Table 1:** **List of qRT-PCR primers**

**Primername**	**Sequence**
Axin 2_s	GCCGACCTCAAGTGCAAACTC
Axin 2_as	GGCTGGTGCAAAGACATAGCC
Gli 1_s	CCTTTAGCAATGCCAGTGACC
Gli 1_as	GAGCGAGCTGGGATCTGTGTAG
Hhip_s	GGCCTCACGACCACATTCTTC
Hhip_as	AGCCATCAGGACCAAAGAGCA
Ptch1_s	TTGGGATCAAGCTGAGTGCTG
Ptch1_as	CGAGCATAGCCCTGTGGTTCT
P21_s	CTGGAGGGCAACTTCGTCTGG
P21_as	GAGTGCAAGACAGCGACAAGG

As shown in the developing spinal cord, activated Wnt/β-catenin signaling increases the expression of repressive Gli3 (Gli3R), which in turn inhibits the Hh pathway [4,11]. We corroborated Gli3R up-regulation upon LiCl treatment in Ptch^+/-^ MB spheres on the protein level (Figures 1c, d, e). However, we cannot judge on the direct regulation of Gli3R via β-catenin. It should be noted that a ternary complex between Gli3R/α-catenin/β-catenin has been observed in chondrocytes, leading to the inhibition of β-catenin transcriptional activity [2,7,17]. In the Ptch^+/-^ MB tumor spheres, we were not able to detect such a complex by co-immunoprecipitation at 8 h of LiCl treatment (data not shown).

**Figure 2:**
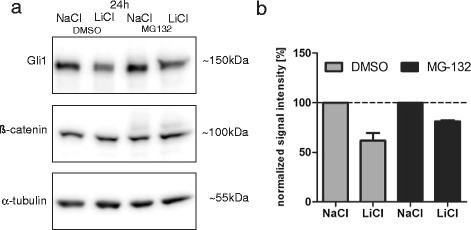
**Gli1 proteasomal degradation is partly reduced with MG-132. (a)** Ptch^+/-^ MB cells were treated with 10 mM NaCl or LiCl for 24 h. 10 μM of proteasome inhibitor MG-132 (A. G. Scientific) or DMSO was added 4.5 h before lysing cells for Western blot analysis. Membranes were probed against Gli1 (R&D), β-catenin (BD Transduction Laboratories, San Jose, CA, USA) and α-tubulin (Sigma-Aldrich). **(b)** Bars represent mean ± s.d. for Gli1 level after 24 h of NaCl or LiCl treatment and 4.5 h treatment with DMSO (grey) or MG-132 (black). The decrease of Gli1 protein levels under LiCl treatment was partly reversed by proteasomal inhibition (*n* = 2, Analysis done in GraphPad Prism 5.01 software).

Although the up-regulation of Gli3R and the concomitant down-regulation of Ptch1, Hhip and Gli1 either on mRNA or protein level fit to the interpretation of Hh pathway inhibition by LiCl-mediated β-catenin stabilization, the mechanism of Gli1 down-regulation remained elusive. To evaluate if proteasomal degradation diminishes Gli1 we investigated the effect on Gli1 protein by the proteasome inhibitor MG-132. LiCl-mediated decrease of Gli1 was partly reversed by proteasomal inhibition (Figures 2a, b). However, the exact mechanism of Gli1 degradation upon β-catenin stabilization requires further investigation.

**Figure 3:**
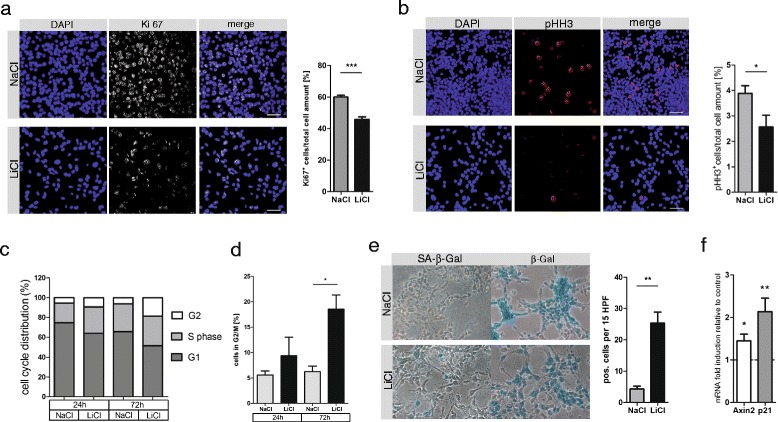
**LiCl treatment decreased MB tumor sphere proliferation and increased senescence. (a)** Primary Ptch^+/-^ MB cells on coverslips (3×10^5^cells/well) were treated for 72 h with 10 mM NaCl or LiCl and stained with rat-anti-Ki67 (DakoCytomation), Alexa-Fluor-568 goat-anti-rat (life technologies) and DAPI (Sigma-Aldrich). Ki67-positive cells were counted (Nikon Eclipse 80i; 20×; NA 0.5), normalized to total cell counts (*n* = 4, mean ± s.d., p-values *** ˂0.0001) and representative pictures were taken by confocal microscopy (Nikon Eclipse C1si; 40×/60×, NA 1.3/1.4; oil). Scale ≙ 50 μm. **(b)** Ptch^+/-^ cells were seeded on μ-Slides/ibi-Treat (ibidi) (8×10^4^cells/well), treated with 10 mM NaCl or LiCl and stained with rabbit-anti-pHH3 (Ser10, Merck Millipore), Alexa-Fluor-568 goat-anti-rabbit (life technologies) and DAPI. pHH3-Positive cells were visualized by confocal microscopy (40×, NA 1.3; oil), counted and normalized to total cell counts. Bars represent mean ± s.d., (*n* = 4, p-value *0.0201). Scale ≙ 50 μm. **(c)** Cell cycle was evaluated by propidiumjodid (5×10^5^ cells; PI, Sigma-Aldrich), measured with a FACS Canto II (Becton Dickinson) and analysed with ModFit LT 3.2 (Verity Software House). Bars represent cell distribution in G2/M, S and G1 phase after 24 h or 72 h of NaCl or LiCl treatment (each 10 mM). **(d)** Bars represent cells in G2/M phase after NaCl or LiCl treatment (mean ± s.d.; *n* = 3, p-value *0.0147). **(e)** LiCl increased SA-βGal-positive Ptch^+/-^ MB cells after 72 h, counted in high power field (40×/0.55NA; Nikon Eclipse TS100). Bars represent mean ± s.d. (*n* = 3, p-value **0.0045). **(f)** Increased senescence of Ptch^+/-^ cells by LiCl associated with increased p21 mRNA levels (see Table 1 for primers). Axin2 served as a control for β-catenin transcription. Dashed line represents NaCl control (*n* = 6, p values left to right: *0.0150, *0.0046; Unpaired student’s t-test in GraphPad Prism 5.01).

Furthermore, LiCl treatment significantly decreased cell proliferation, indicated by diminished Ki67-positive cells (Figure 3a) and by decreased cells marked by the more specific mitotic marker phospho-histone H3 (pHH3; Figure 3b). Hence, LiCl treatment led to an accumulation of cells in G2/M cell cycle phase (Figures 3c, d). As shown recently, G2/M accumulation also occurs in LiCl-treated endometrial and glioma cancer cell lines [13,14], and LiCl treatment of pancreatic ductal adenocarcinomas reduces the tumorigenicity of cells through Hh inhibition [15].

Along this line, here we show that in primary Ptch^+/-^ MB cells LiCl treatment increased the number of senescence-associated β-galactosidase (SA-βGal)-positive cells (Figure 3e) and upregulated p21 after 72 h as a senescence marker (Figure 3f). These findings suggest that LiCl induces a senescent-like state in MB tumor cells. Apoptosis however, was not detectable after 8 h, 24 h and 72 h of LiCl treatment investigated by annexin V staining (data not shown). Furthermore, LiCl treatment did not specifically increase autophagy in Ptch^+/-^ MB spheres, evidenced by staining for LC3 (data not shown). However this aspect requires further investigation.

**Figure 4:**
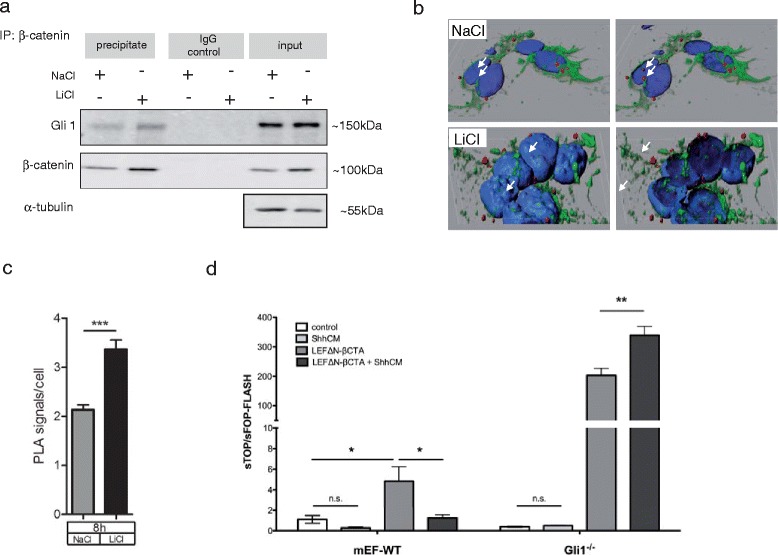
**Gli1 interacts with β-catenin in Ptch** ^**+/-**^ **MB tumor spheres. (a)** Primary Ptch^+/-^ MB cells were treated with NaCl or LiCl (8 h) and the extracts were immunoprecipitated with an anti-β-catenin antibody (BD Transduction Laboratories) prebound to protein G-agarose (Roche, Basel, Switzerland). Co-precipitated Gli1 (R&D Systems) was verified by Western blotting. **(b)** For PLA, primary Ptch^+/-^ MB cells were seeded on μ-slides (ibidi), treated with NaCl or LiCl for 8 h, incubated with anti-Gli1 (R&D Systems) and anti-β-catenin antibodies (BD Transduction Laboratories), corresponding anti-goat (minus) or anti-mouse (plus) PLA probes and ligation-mix was added (Duolink In Situ, Detection Kit orange; Excitation: 554 nm, Emission: 579 nm, Sigma-Aldrich). Samples were examined by confocal microscopy (Nikon Eclipse C1si; 40×/60×, NA 1.3/1.4; oil; 50pictures/condition). Amaris (7.7; Bitplane, Switzerland) was used to generate surface-rendered nuclei from 60× images; a clipping plane was introduced to visualize nuclear PLA signals. Co-immunoprecipitation and PLA revealed an interaction between β-catenin and Gli1, which increases under LiCl treatment. **(c)** Total number of interactions (red dots; protein-protein distance ≤ 40 nm) was normalized to the total number of DAPI-positive nuclei per image. Bars represent PLA signals/cell after 8 h treatment with NaCl (grey) and LiCl (black). Analysis was done with GraphPad Prism 5.01 software (p-value 0.0004). **(d)** 5×10^4^ mEF-WT and mEF-Gli1^-/-^ cells were seeded per 24-well and transfected after 24h with either 8xSuperTOP or 8xSuperFOP (380 ng), TK-Renilla (20 ng) and LEFΔN-βCTA (100 ng) or empty vector pcDNA3.1 (100 ng) using 4 μl Lipofectamine LTX with 0.5 μl PLUS reagent (Life Technology) in Optimem (Life Technology). 24 h post transfection cells were treated with Shh-conditioned medium (ShhCM) for 24 h. Luciferase activity was measured on a TECAN plate reader using the Dual Luciferase Kit (Promega). (*n* = 5), p-values left to right: * 0.0331, *0.0372, **0.0075.

We next analyzed the downstream effects of GSK3 inhibition with respect to Wnt/β-catenin and Hh crosstalk, as β-catenin interaction with Gli1 has previously been suggested to occur in endometrial carcinoma in which both factors cooperate to drive tumor growth [18]. To this end we immunoprecipitated β-catenin from Ptch^+/-^ MB cultures after 8 h LiCl stimulation and blotted for Gli1, which was significantly increased upon β-catenin stabilization by LiCl, although low amounts of co-precipitation was also detectable in the NaCl controls (Figure 4a). This suggests that stabilized β-catenin is able to sequester Gli1, making Gli1 unavailable for transcriptional activity and likely subjecting it to protein degradation.

To visualize the β-catenin-Gli1 interaction upon LiCl stimulation, we performed a proximity ligation assay (PLA) with antibodies against β-catenin and Gli1. Indeed, this approach corroborated the β-catenin-Gli1 interaction observed in co-precipitation experiments (Figures 4b, c, Additional file 4: Figure S4). Furthermore, 3D reconstruction of confocal Z-stacks revealed the localization of a small fraction of β-catenin-Gli1 within the nucleus, whereas most of the complex localized to the cytoplasm of Ptch^+/-^ MB tumor cells (Figure 4b). This observation suggests that β-catenin might sequester Gli1 in the nucleus as well as in the cytoplasm, thus inhibiting Gli1-mediated transcription. As published previously in tongue taste papilla turnover, in squamous cell carcinoma and in gastric cancer, the Hh pathway can equally inhibit β-catenin transcriptional activity [4,19].

Taking advantage of the putative mutual inhibition of Gli1 and β-catenin and to further analyze the specific role of Gli1 for functional interaction with β-catenin, we explored the inhibitory function of Shh on β-catenin signaling using WT as well as Gli1^-/-^ mouse embryonic fibroblasts (MEFs). MEFs were transfected with the chimeric construct LEFΔN-βCTA, conferring dominant β-catenin-LEF transcription, and subsequently stimulated with control or Shh-conditioned medium (ShhCM) [20]. As a read-out we utilized the superTOP/FOP-FLASH assay, measuring luciferase activity driven by a LEF-responsive promotor [21]. Interestingly, the inhibitory function of ShhCM on β-catenin transcription was completely abrogated in Gli1^-/-^ MEFs (Figure 4d), suggesting that the interaction of Gli1 with LEFΔN-βCTA is required for transcriptional inhibition. As the LEFΔN-βCTA construct contains only the C-terminal transactivation domain of β-catenin (amino acid 695-781), these findings are suggestive that the C-terminus of β-catenin is crucial for the interaction with Gli1. It will be interesting to analyze in detail which exact binding-domains of β-catenin and Gli1 are required for protein-protein interaction.

Furthermore, we investigated the role of β-catenin for hedgehog-mediated MB sphere growth by knocking down β-catenin by a siRNA approach. Interestingly, knock-down of β-catenin abrogated the growth inhibitory function of LiCl treatment (Additional file 5: Figure S5). Together these findings suggest that β-catenin stabilization and availability is crucial for LiCl-induced Hh pathway inhibition via the sequestration of Gli1.

Finally, in order to understand if β-catenin activation might be beneficial for MB patients, we transplanted primary mouse Ptch^+/-^ MB cells subcutaneously into the flanks of NMRI/nude mice and treated the animals with either LiCl (7,5 μl/g body weight, 1.2 M) or NaCl (7,5 μl/g body weight, 1.2 M) as control [9,22].

**Figure 5:**
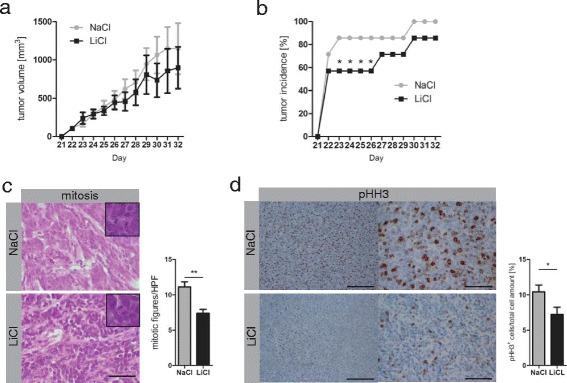
**LiCl treatment delayed growth and decreased volume of Ptch** ^**+/-**^ **MB-derived tumors. (a)** 1×10^6^ cells in 100 μl PBS were subcutaneously injected into HsdCpb:NMRI-Foxn1nu mice (6-8weeks, Harlan Laboratories) and animals were treated with LiCl (7,5 μl/g body weight, 1.2 M) or NaCl (7,5 μl/g body weight, 1.2 M) from day 5 post-transplantation. From day 21 tumors were measured daily thereafter (electronic disk micrometers, Vogel Germany), the mean of tumor volume was determined by the formula: π/6× maximal-diameter x minimal-diameter^2^ (mm^3^) until animals were sacrificed by day 32. **(b)** Dots represent the percentage of tumor-bearing animals. Statics were performed using PSPP 0.8.2 as described previously [23]. Equal numbers (n = 7) from NaCl- or LiCl-treated mice were tested for tumor formation probability by a non-parametric one-tailed binomial proportions test, setting the expected outcome for tumor formation to 90%. There was no significant difference between the expectation and the tumor incidence for the NaCl-treated group (p-value 0.52), but for the LiCl-treated group (p-value *0.03). **(c)** Paraffin sections (3 μm) of tumors were stained with hematoxylin and eosin, mitotic figures were counted per high power field and pictures were taken at an Olympus BX50 microscope 40× (NA 0.75). Bars represent mean ± s.d. (*n* = 7 (NaCl), *n = 6* (LiCl)), p-value **0.0021). Scale bar ≙ 50 μm. Analysis was done with GraphPad Prism 5.01. **(d)** Immunohistochemistry with anti-pHH3 (Merck Millipore) antibody on paraffin sections (3 μm) was performed using Ventana Discovery XT system (Ventana, USA), examined with the Axiophot (Zeiss, Germany, Achroplan 0.65) and analyzed in Stereo Investigator 4.34 (MicroBright Field.Inc Europe, Germany). Pictures were taken at a Nikon Eclipse 80i (Nikon, Japan; 10×/40×; NA 0.5). Bars represent mean ± s.d. (*n* = 7 (NaCl), *n* = 6 (LiCl), p-value *0.0463). Scale bar represent 200 μm (left), 50 μm (right).

LiCl treatment resulted in a significant delay in initial tumor incidence that we defined as “tumor take”, ranging between day 22 and 26 (Figure 5b). Nevertheless, nearly all animals, irrespective of the treatment, developed tumors by day 32, with a tendency of smaller tumors in the LiCl group (Figures 5a, b). Taking together this finding suggests that LiCl treatment significantly inhibits tumor take (Figure 5b), although we did not observe a significantly reduced tumor burden at the experimental endpoint.

However, we observed reduced mitotic figures in LiCl-treated tumors, which was supported by decreased phospho-histone H3 (pHH3)-positive cells (Figures 5c, d). This reflects a reduced population of cells in M phase and suggests that the majority of cells are arrested in G2 phase, corroborating our *in vitro* findings (Figure 3b). Apoptosis was not altered in LiCl-treated MB tumors, evidenced by cleaved caspase 3 staining (Additional file 6: Figures S6 a, b).

It is interesting however, that LiCl tumors showed decreased necrotic areas (data not shown), although tumor size did not significantly differ from controls. In this regard it is worth mentioning that we recently reported on a normalizing effect of β-catenin activation in endothelial cells of glioma vasculature [24]. This would support the hypothesis that LiCl treatment of subcutaneous MB tumors might affect both, cell proliferation as well as tumor microenvironment by normalizing the vasculature. When we analyzed MB tumors for vessel density and smooth muscle cell coverage by CD31 and αSMA staining, respectively, we did not observe significant differences between the NaCl- and the LiCl-treated group (Additional file 6: Figure S6 c, d). This result might be explained by the different tumor models used in the study of Reis et al. and the present manuscript. Glioblastoma are well known to be highly angiogenic, whereas MB does not strongly induce vessel growth [25]. This requires more in depth evaluation, also at different stages of tumor growth that is however beyond the scope of this study.

The overall tumor histopathology of the subcutaneous Ptch^+/-^ tumors did not differ with respect to extracellular matrix and GFAP staining (Additional file 7: Figure S7).

The systemic treatment with LiCl and the resulting stabilization of β-catenin will always target more cell types then the tumor cell itself. Consequently, we have to estimate the benefit of the treatment by the net outcome of tumor burden. It should be noted that the clinical impact of these findings might relate to some types of Shh-driven MB that occur in infants and young adults, which are difficult to treat with established regimes or by Smo inhibitors that are currently in clinical trials [26]. It seems to be common to these treatment-resistant MBs that they carry mutations downstream of Ptch or Smo in genes such as suppressor of fused (SuFu) or Gli2. Herein the stabilization of β-catenin at an early time point of the disease might have beneficial effects by directly targeting Gli1 and by supporting the chemotherapeutic treatment of the tumor cells. It should be noted that it is likely that human Shh-driven MB, harboring a mutation in Ptch, also respond to β-catenin activation with reduction of Gli1. However, more work is required to evaluate the translational potential of our finding.

In summary our data show for the first time that activation of Wnt/β-catenin signaling by LiCl treatment inhibits proliferation of Shh-driven mouse MB via the interaction of β-catenin and Gli1.

This supports the concept that Gli1 interacts with β-catenin by default, and that an increase of stabilized β-catenin is able to sequester an increased amount of Gli1 and vice versa. This scheme of a balanced interaction implies that β-catenin stabilization could be titrated to a level at which virtually all Gli1 becomes sequestered, without affecting canonical Wnt pathway activation. These findings may open novel possibilities for therapeutic interventions for MB patients.

## Additional files

Additional file 1: Figure S1. LiCl treatment reduced tumor cell self-renewal and growth. (a) Ptch^+/-^ MB cells were cultivated for one week with either 10 mM NaCl or LiCl, growth factors and NaCl/LiCl were added every third day. After 7 days tumor spheres were counted, dissociated mechanically and subsequently seeded for a second and third sphere forming clonal assay (CA1 - 3). Ptch^+/-^ MB cells were kept on either NaCl or LiCl to investigate if Ptch^+/-^ MB cells might adapt to the LiCl treatment. p-Values left to right: **0.0020, ***0.0006, *0.0104. Ptch^+/-^ MB cells seeded for three subsequent clonal assays (CA) showed no adaption to the LiCl treatment. (b) Primary Ptch^+/-^ MB cells were seeded on fibronectin/poly-L-ornithin-coated 96-well plates (1×10^4^cells/well) and cultured overnight. Cells were treated with descending concentrations of NaCl or LiCl for 72 h and stained with crystal violet. Absorbance at 595 nm was measured (TECAN reader infinite M200 pro, TECAN, Männedorf, Switzerland) and crystal violet background staining was subtracted. Bars represent mean ± s.d., (*n* = 4), p-values left to right: ** 0.0098, *0.0478. Additional file 2: Figure S2. β-Catenin stabilization by LiCl treatment increased Axin2 protein level. (a) Primary Ptch^+/-^ MB spheres were treated with 10 mM NaCl or LiCl and harvested after 24 h and 72 h. Membranes were probed with antibodies against Axin2 (Abcam) and α-tubulin (Sigma-Aldrich) as a loading control. Bars represent mean ± s.d of Axin2 protein level after 24 h (grey) and 72 h (black) (*n* = 4, p-value *0.0164). Additional file 3: Figure S3. β-Catenin stabilization by 6BIO treatment decreased Gli1 protein level and pHH3 positive cells. (a) Primary Ptch^+/-^ MB spheres were treated with 10 μM 6BIO or DMSO as control, harvested after 72 h and lysed and separated in cytoplasmic (C) and nuclear fraction (N). Membranes were probed with antibodies against Gli1 (R&D Systems) and β-catenin (BD Transduction Laboratories). Lamin B1 (Abcam) and α-tubulin (Sigma-Aldrich) served as loading controls. Gli1 protein levels decreased, β-catenin protein levels increased under 6BIO treatment. (b) Primary Ptch^+/-^ MB cells were seeded on μ-Slide 8 well ibi-Treat slides (ibidi, Martinsried, Germany) (8x10^4^cells/well) and treated with 10 μM 6BIO or DMSO as control. Immunocytochemistry was performed with rabbit-anti-pHH3 (Ser10) antibody (Merck Millipore) followed by Alexa-Fluor-56 goat-anti-rabbit antibody (life technologies) and DAPI. pHH3-positive cells were counted at a confocal microscope (Nikon Eclipse C1si; 40×, NA 1.3; oil immersion and normalized to total cell count. Bars represent mean ± s.d., (*n* = 3, p-value *** < 0.0001). Scale bar represents 50 μm. The number of pHH3 positive cells decreased under treatment with 6BIO. Additional file 4: Figure S4. Increased interaction of β-Catenin with Gli1 upon LiCl stimulation. For PLA, primary Ptch^+/-^ MB cells were seeded on μ-slides (ibidi, Martinsried, Germany) and treated with NaCl or LiCl for 8 h. Cells were incubated with antibodies against Gli1 (R&D Systems) and β-catenin (BD Transduction Laboratories) and corresponding anti-goat (minus) or anti-mouse (plus) PLA probes. Ligation-mix, consisting of two oligonucleotides, and amplification-mix, consisting of nucleotides and fluorescently labeled oligonucleotides, was added (Duolink In Situ, Detection Kit orange (Excitation: 554 nm, Emission: 579 nm, Sigma-Aldrich). Samples were examined by confocal microscopy (Nikon Eclipse C1si; 40×/60×, NA 1.3/1.4; oil immersion; 50 pictures/condition). Additional file 5: Figure S5. β-catenin knock down with siRNA abrogated the growth inhibitory function of LiCl treatment. Primary Ptch^+/-^ cells were transfected with either 5nM or 10nM of Silencer®Select Pre-designed siRNA against β-catenin or a control#1 siRNA (Ambion, life technologies), MetafectenePro was used as transfection reagent and incubated for 5 h. (a) 24 h post transfection cells (5nM and 10nM) were treated with either NaCl or LiCl for 72 h. 72 h of LiCl treatment led to decreased cell growth when cells were transfected with control siRNA but had no effect on β-catenin knock down cells (b) 24 h and 48 h post transfection a >70% knock-down of β-catenin was examined by qRT-PCR. Additional file 6: Figure S6. Treatment of nude mice with NaCl or LiCl did not change vessel density, amount of cleaved caspase positive cells or αSMA positive cells. Immunohistochemistry with (a) anti-cleaved Caspase 3 (Cell Signaling) on paraffin-embedded sections (3 μm) of flank tumors or (b) anti-cleaved Caspase 3 (Cell signaling) on paraffin-embedded cell pellets and (d) anti-CD31 antibody (Clone SZ31, Dianova, Hamburg, Germany) was performed using the automated Ventana Discovery XT staining system (Ventana, Tucson, Arizona USA) and standard protocols. Slides were examined with the Axiophot light Microscope (Zeiss, Germany, Achroplan 0.65) and analyzed with the Stereo Investigator Software 4.34 (MicroBright Field.Inc Europe, Magdeburg, Germany). Pictures were taken at a light microscope (Nikon Eclipse 80i; Nikon, Japan; 10×/40×; NA 0.5). Bars represent mean ± s.d. for NaCl (grey) or LiCl (black) treatment (CD31: *n* = 6 (NaCl), *n* = 6 (LiCl); cleaved Caspase 3: *n* = 6 (NaCl), *n* = 4 (LiCl); cleaved Caspase 3 cell pellet: *n* = 1 (/NaCl and LiCl)). Scale bar represent 200 μm (left) and 50 μm (right). (c) Immunofluorescence staining with anti-α-smooth muscle actin-Cy3 antibody was performed on paraffin-embedded sections (3 μM). Staining intensity was measured with ImageJ 1.47v software and normalized to tumor size. Additional file 7: Figure S7. Histo-pathological characterization of subcutaneous Ptch^+/-^ tumors. Paraffin-embedded sections (3 μm) of flank tumors were stained with hematoxylin and eosin. Immunohistochemistry with anti-GFAP antibody (DakoCytomation) and reticulin (special stain Ventana) on paraffin-embedded sections (3 μm) of flank tumors was performed using the automated Ventana Discovery XT staining system (Ventana, Tucson, Arizona USA) and standard protocols. Stainings were visualized by Olympus BX50 light microscope 10× (NA 0.30). Scale bar represent 200 μm.

## Abbreviations

Shh: Sonic hedgehog
MB: Medulloblastoma
Ptch^+/-^: Patched heterozygous
Hh: Hedgehog
Ptch1: Patched1
6BIO: (2’Z,3’E)-6-Bromoindirubin-3’-oxime
MEFs: Mouse embryonic fibroblasts
PLA: Proximity ligation assay
ShhCM: Sonic hedgehog-conditioned medium
pHH3: Phospho-Histone H3
LiCl: Lithium-chloride
NaCl: Sodium-chloride

## References

[1] Liu H, Fergusson MM, Castilho RM, Liu J, Cao L, Chen J (2007). Augmented Wnt signaling in a mammalian model of accelerated aging. Science.

[2] Taylor MD, Northcott PA, Korshunov A, Remke M, Cho Y-J, Clifford SC (2012). Molecular subgroups of medulloblastoma: the current consensus. Acta Neuropathol.

[3] Liu H, MacCallum D, Edwards C, Gaffield W, Mistretta C (2004). Sonic hedgehog exerts distinct, stage-specific effects on tongue and taste papilla development. Dev Biol.

[4] Schneider FT, Schänzer A, Czupalla CJ, Thom S, Engels K, Schmidt MHH (2010). Sonic hedgehog acts as a negative regulator of {beta}-catenin signaling in the adult tongue epithelium. Am J Pathol.

[5] Pöschl J, Bartels M, Ohli J, Bianchi E, Kuteykin-Teplyakov K, Grammel D (2014). Wnt/β-catenin signaling inhibits the Shh pathway and impairs tumor growth in Shh-dependent medulloblastoma. Acta Neuropathol.

[6] Anne SL, Govek E-E, Ayrault O, Kim JH, Zhu X, Murphy DA (2013). WNT3 inhibits cerebellar granule neuron progenitor proliferation and medulloblastoma formation via MAPK activation. PLoS One.

[7] Northcott PA, Jones DTW, Kool M, Robinson GW, Gilbertson RJ, Cho Y-J (2012). Medulloblastomics: the end of the beginning. Nat Rev Cancer.

[8] Goodrich L, Milenkovic L, Higgins K, Scott M (1997). Altered neural cell fates and medulloblastoma in mouse patched mutants. Science.

[9] Mueller S, Chang S (2009). Pediatric brain tumors: current treatment strategies and future therapeutic approaches. Neurotherapeutics.

[10] Clevers H (2006). Wnt/beta-catenin signaling in development and disease. Cell.

[11] Alvarez-Medina R, Cayuso J, Okubo T, Takada S, Martí E (2008). Wnt canonical pathway restricts graded Shh/Gli patterning activity through the regulation of Gli3 expression. Development.

[12] Ingham PW (2008). Hedgehog signalling. Curr Biol.

[13] Yin Y, Kizer NT, Thaker PH, Chiappinelli KB, Trinkaus KM, Goodfellow PJ (2013). Glycogen synthase kinase 3β inhibition as a therapeutic approach in the treatment of endometrial cancer. Int J Mol Sci.

[14] Nowicki MO, Dmitrieva N, Stein AM, Cutter JL, Godlewski J, Saeki Y (2008). Lithium inhibits invasion of glioma cells; possible involvement of glycogen synthase kinase-3. Neuro Oncol.

[15] Peng Z, Ji Z, Mei F, Lu M, Ou Y, Cheng X (2013). Lithium inhibits tumorigenic potential of PDA cells through targeting hedgehog-GLI signaling pathway. PLoS One.

[16] Meijer L, Flajolet M, Greengard P (2004). Pharmacological inhibitors of glycogen synthase kinase 3. Trends Pharmacol Sci.

[17] Rhee J, Ryu JH, Kim JH, Chun CH, Chun JS (2012). α-Catenin inhibits β-catenin-Tcf/Lef transcriptional activity and collagen type II expression in articular chondrocytes through formation of a Gli3R/α-catenin/β-catenin ternary complex. J Biol Chem.

[18] Liao X, Siu MK, Au CW, Chan QK, Chan HY, Wong ES (2009). Aberrant activation of hedgehog signaling pathway contributes to endometrial carcinogenesis through. Mod Pathol.

[19] Kim J-H, Shin HS, Lee SH, Lee I, Lee YS, Park JC (2010). Contrasting activity of Hedgehog and Wnt pathways according to gastric cancer cell differentiation: relevance of crosstalk mechanisms. Cancer Sci.

[20] Vleminckx K, Kemler R, Hecht A (1999). The C-terminal transactivation domain of beta-catenin is necessary and sufficient for signaling by the LEF-1/beta-catenin complex in Xenopus laevis. Mech Dev.

[21] Veeman MT, Slusarski DC, Kaykas A, Louie SH, Moon RT (2003). Zebrafish prickle, a modulator of noncanonical Wnt/Fz signaling, regulates gastrulation movements. Curr Biol.

[22] Schou M (1997). Forty years of lithium treatment. Arch Gen Psychiatry.

[23] Walter CJ, Bell LTO, Parsons SR, Jackson C, Borley NR, Wheeler JMD (2013). Prevalence and significance of anaemia in patients receiving long-course neoadjuvant chemoradiotherapy for rectal carcinoma. Colorectal Dis.

[24] Reis M, Czupalla CJ, Ziegler N, Devraj K, Zinke J, Seidel S (2012). Endothelial Wnt/β-catenin signaling inhibits glioma angiogenesis and normalizes tumor blood vessels by inducing PDGF-B expression. J Exp Med.

[25] Reis M, Liebner S (2013). Wnt signaling in the vasculature. Exp Cell Res.

[26] Kool M, Jones DTW, Jäger N, Northcott PA, Pugh TJ, Hovestadt V (2014). Genome sequencing of SHH medulloblastoma predicts genotype-related response to smoothened inhibition. Cancer Cell.

