# Supplementary figures and images for: β-Catenin-Gli1 interaction regulates proliferation and tumor growth in medulloblastoma
Source: Mol Cancer. 2015 Feb 3;14(1):17. doi: 10.1186/s12943-015-0294-4 (PMC4320815; doi:10.1186/s12943-015-0294-4)

Suppl. Fig. 1

a

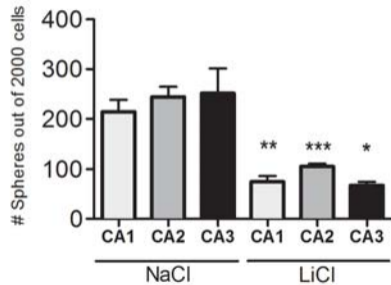

b

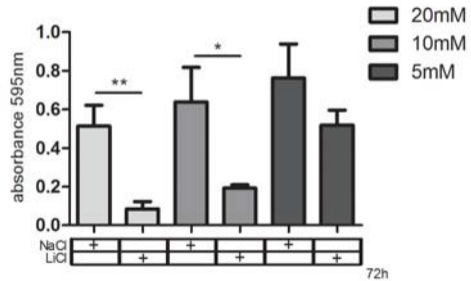

Supplement: Additional file 1: Figure S1. — LiCl treatment reduced tumor cell self-renewal and growth. (a) Ptch+/- MB cells were cultivated for one week with either 10 mM NaCl or LiCl, growth factors and NaCl/LiCl were added every third day. After 7 days tumor spheres were counted, dissociated mechanically and subsequently seeded for a second and third sphere forming clonal assay (CA1 - 3). Ptch+/- MB cells were kept on either NaCl or LiCl to investigate if Ptch+/- MB cells might adapt to the LiCl treatment. p-Values left to right: **0.0020, ***0.0006, *0.0104. Ptch+/- MB cells seeded for three subsequent clonal assays (CA) showed no adaption to the LiCl treatment. (b) Primary Ptch+/- MB cells were seeded on fibronectin/poly-L-ornithin-coated 96-well plates (1×10^4^cells/well) and cultured overnight. Cells were treated with descending concentrations of NaCl or LiCl for 72 h and stained with crystal violet. Absorbance at 595 nm was measured (TECAN reader infinite M200 pro, TECAN, Männedorf, Switzerland) and crystal violet background staining was subtracted. Bars represent mean ± s.d., (n = 4), p-values left to right: ** 0.0098, *0.0478. [file 12943_2015_294_MOESM1_ESM.pdf]

## Suppl. Fig. 2

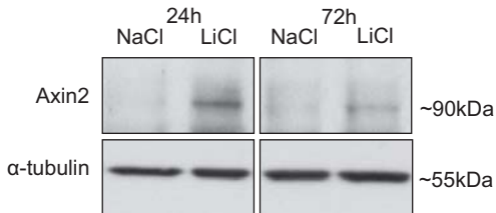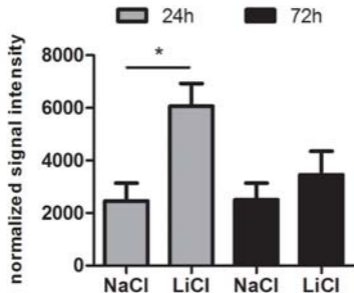

Supplement: Additional file 2: Figure S2. — β-Catenin stabilization by LiCl treatment increased Axin2 protein level. (a) Primary Ptch+/- MB spheres were treated with 10 mM NaCl or LiCl and harvested after 24 h and 72 h. Membranes were probed with antibodies against Axin2 (Abcam) and α-tubulin (Sigma-Aldrich) as a loading control. Bars represent mean ± s.d of Axin2 protein level after 24 h (grey) and 72 h (black) (n = 4, p-value *0.0164). [file 12943_2015_294_MOESM2_ESM.pdf]

Suppl. Fig. 3

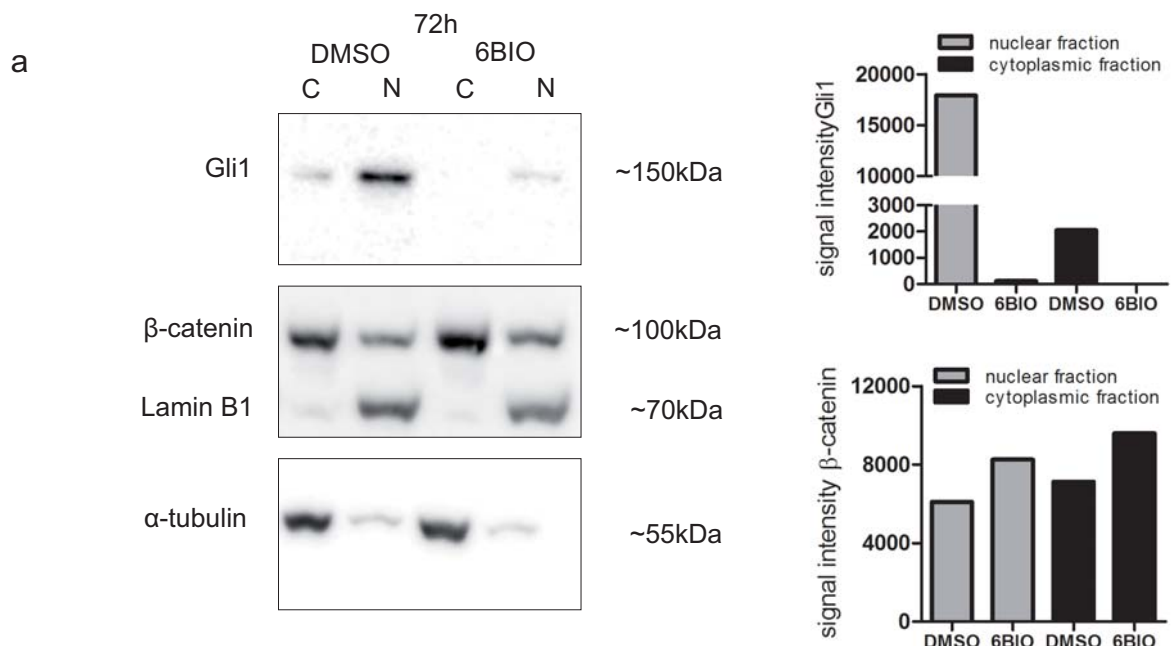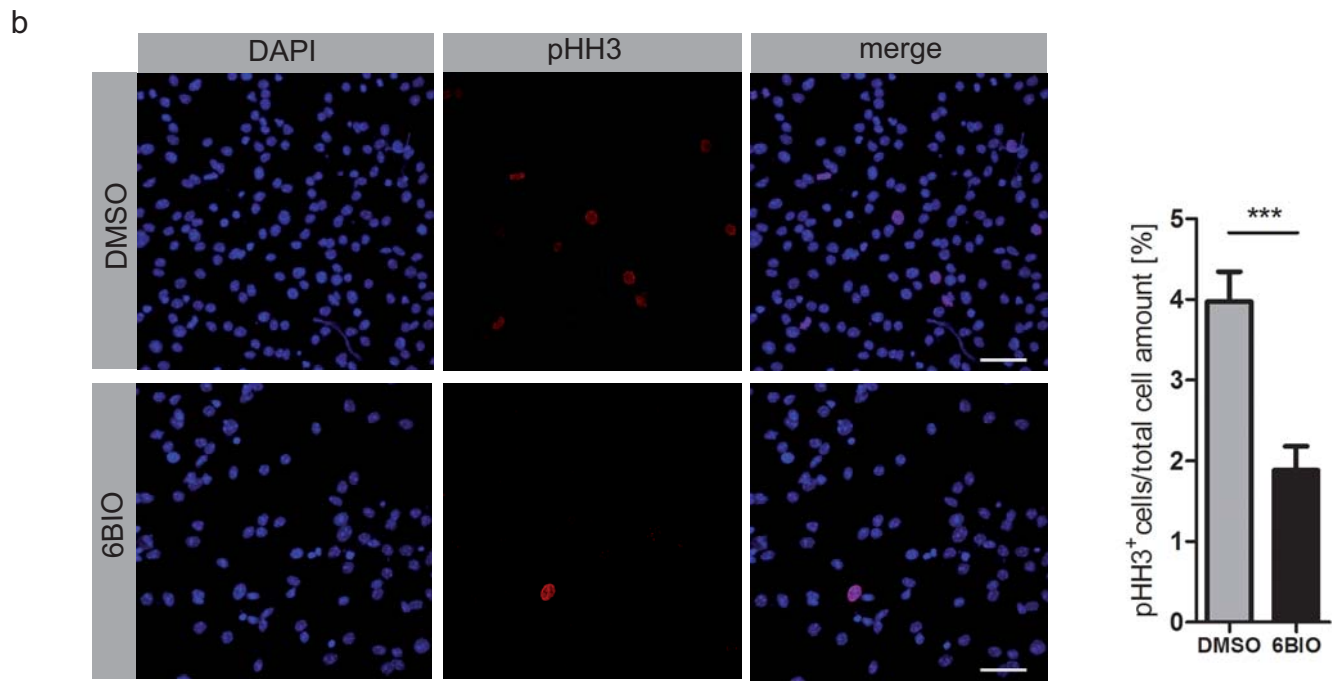

Supplement: Additional file 3: Figure S3. — β-Catenin stabilization by 6BIO treatment decreased Gli1 protein level and pHH3 positive cells. (a) Primary Ptch+/- MB spheres were treated with 10 μM 6BIO or DMSO as control, harvested after 72 h and lysed and separated in cytoplasmic (C) and nuclear fraction (N). Membranes were probed with antibodies against Gli1 (R&D Systems) and β-catenin (BD Transduction Laboratories). Lamin B1 (Abcam) and α-tubulin (Sigma-Aldrich) served as loading controls. Gli1 protein levels decreased, β-catenin protein levels increased under 6BIO treatment. (b) Primary Ptch+/- MB cells were seeded on μ-Slide 8 well ibi-Treat slides (ibidi, Martinsried, Germany) (8x10^4^cells/well) and treated with 10 μM 6BIO or DMSO as control. Immunocytochemistry was performed with rabbit-anti-pHH3 (Ser10) antibody (Merck Millipore) followed by Alexa-Fluor-56 goat-anti-rabbit antibody (life technologies) and DAPI. pHH3-positive cells were counted at a confocal microscope (Nikon Eclipse C1si; 40×, NA 1.3; oil immersion and normalized to total cell count. Bars represent mean ± s.d., (n = 3, p-value *** < 0.0001). Scale bar represents 50 μm. The number of pHH3 positive cells decreased under treatment with 6BIO. [file 12943_2015_294_MOESM3_ESM.pdf]

# Suppl. Fig. 4

a

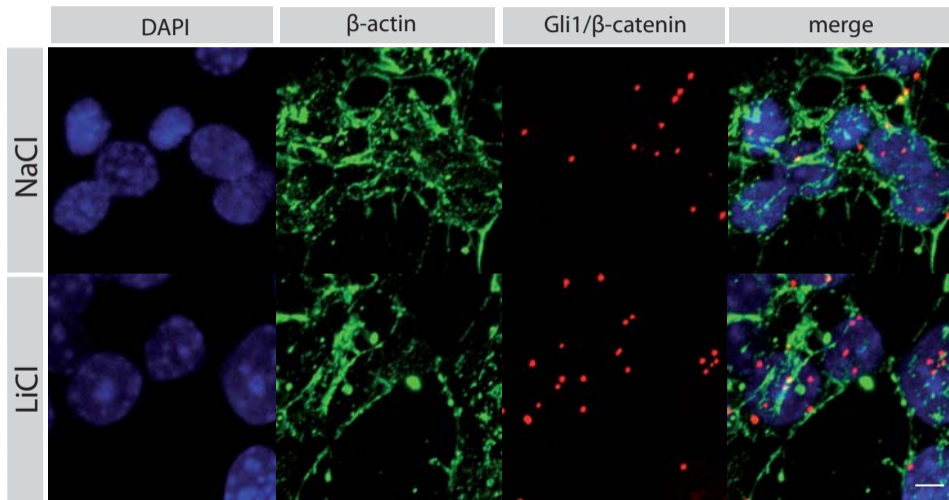

Supplement: Additional file 4: Figure S4. — Increased interaction of β-Catenin with Gli1 upon LiCl stimulation. For PLA, primary Ptch+/- MB cells were seeded on μ-slides (ibidi, Martinsried, Germany) and treated with NaCl or LiCl for 8 h. Cells were incubated with antibodies against Gli1 (R&D Systems) and β-catenin (BD Transduction Laboratories) and corresponding anti-goat (minus) or anti-mouse (plus) PLA probes. Ligation-mix, consisting of two oligonucleotides, and amplification-mix, consisting of nucleotides and fluorescently labeled oligonucleotides, was added (Duolink In Situ, Detection Kit orange (Excitation: 554 nm, Emission: 579 nm, Sigma-Aldrich). Samples were examined by confocal microscopy (Nikon Eclipse C1si; 40×/60×, NA 1.3/1.4; oil immersion; 50 pictures/condition). [file 12943_2015_294_MOESM4_ESM.pdf]

Suppl. Fig. 5

a

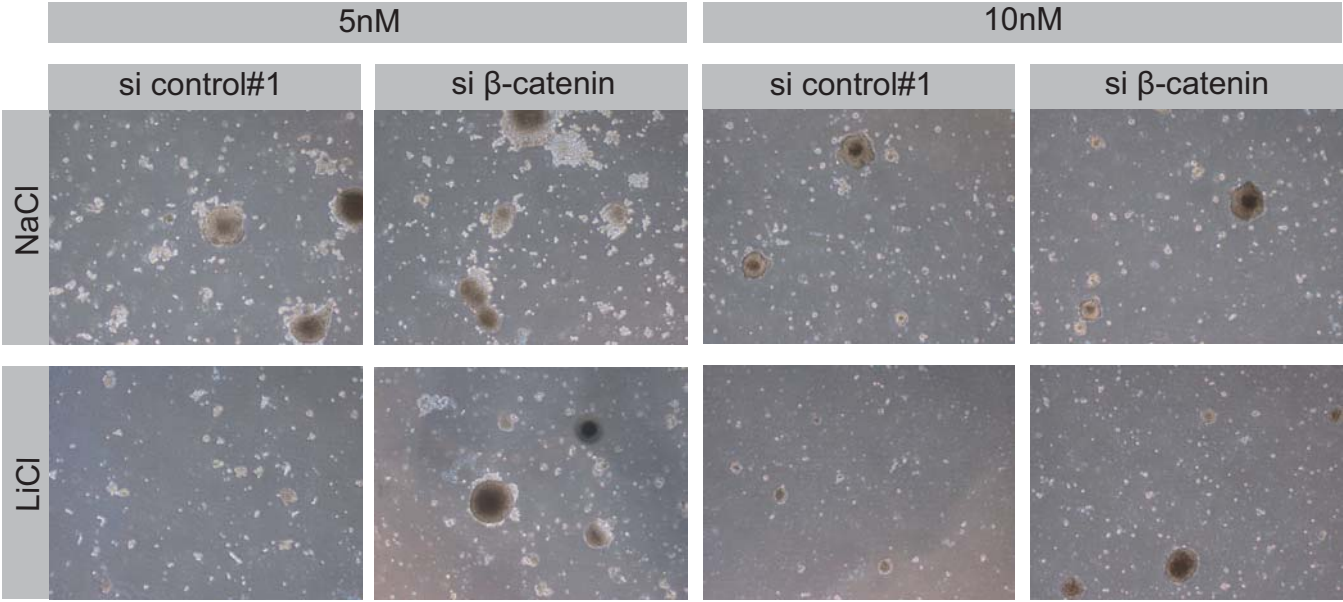

b

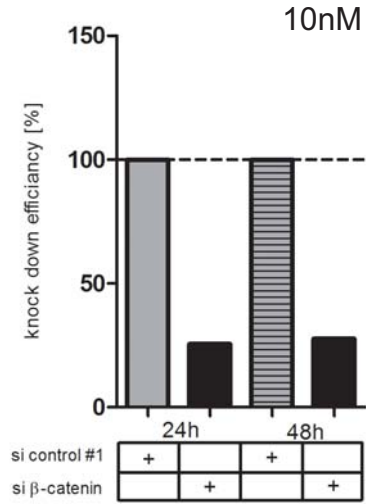

Supplement: Additional file 5: Figure S5. — β-catenin knock down with siRNA abrogated the growth inhibitory function of LiCl treatment. Primary Ptch+/- cells were transfected with either 5nM or 10nM of Silencer®Select Pre-designed siRNA against β-catenin or a control#1 siRNA (Ambion, life technologies), MetafectenePro was used as transfection reagent and incubated for 5 h. (a) 24 h post transfection cells (5nM and 10nM) were treated with either NaCl or LiCl for 72 h. 72 h of LiCl treatment led to decreased cell growth when cells were transfected with control siRNA but had no effect on β-catenin knock down cells (b) 24 h and 48 h post transfection a >70% knock-down of β-catenin was examined by qRT-PCR. [file 12943_2015_294_MOESM5_ESM.pdf]

a

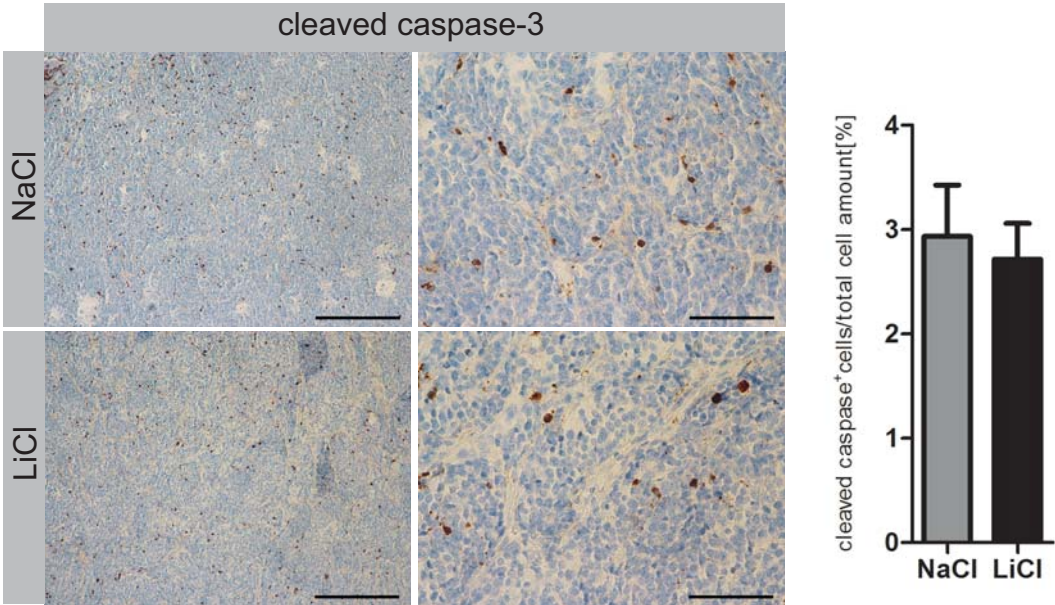

b

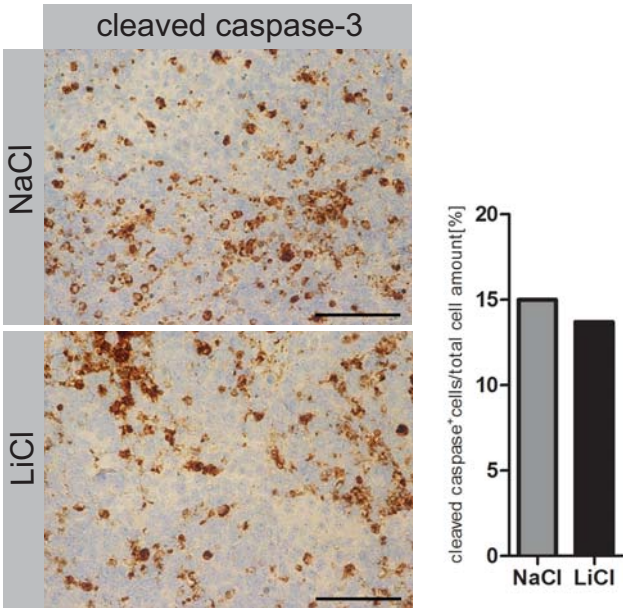

c

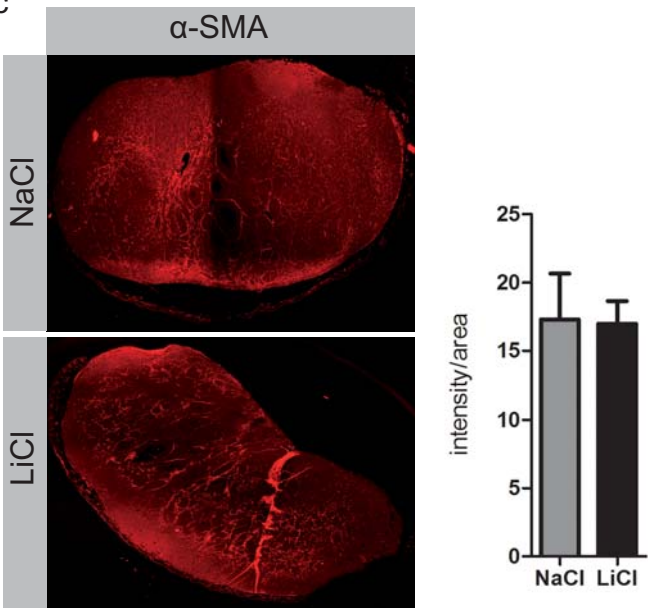

d

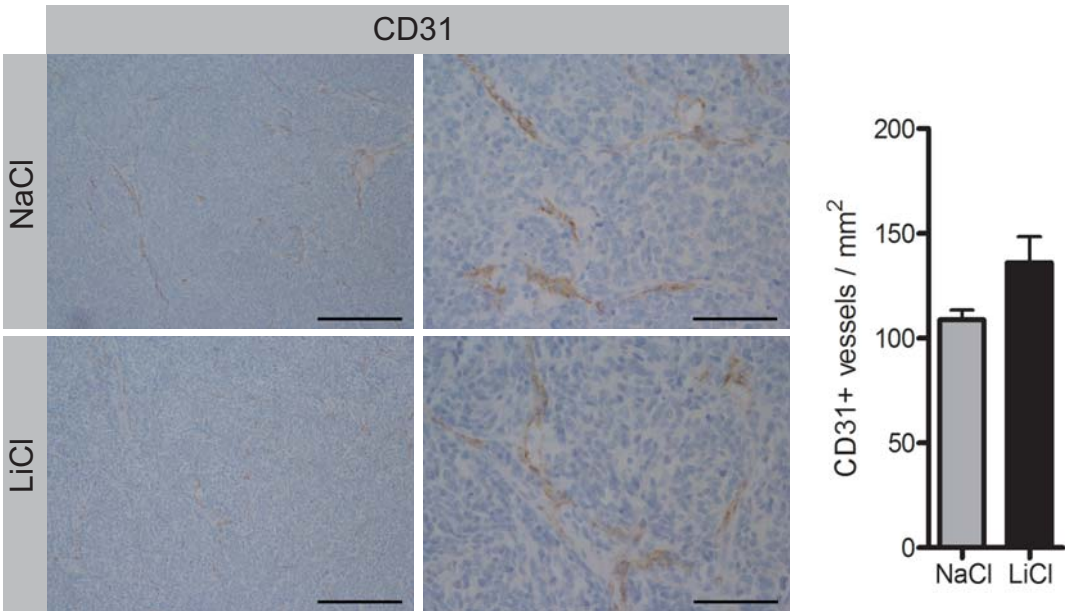

Supplement: Additional file 6: Figure S6. — Treatment of nude mice with NaCl or LiCl did not change vessel density, amount of cleaved caspase positive cells or αSMA positive cells. Immunohistochemistry with (a) anti-cleaved Caspase 3 (Cell Signaling) on paraffin-embedded sections (3 μm) of flank tumors or (b) anti-cleaved Caspase 3 (Cell signaling) on paraffin-embedded cell pellets and (d) anti-CD31 antibody (Clone SZ31, Dianova, Hamburg, Germany) was performed using the automated Ventana Discovery XT staining system (Ventana, Tucson, Arizona USA) and standard protocols. Slides were examined with the Axiophot light Microscope (Zeiss, Germany, Achroplan 0.65) and analyzed with the Stereo Investigator Software 4.34 (MicroBright Field.Inc Europe, Magdeburg, Germany). Pictures were taken at a light microscope (Nikon Eclipse 80i; Nikon, Japan; 10×/40×; NA 0.5). Bars represent mean ± s.d. for NaCl (grey) or LiCl (black) treatment (CD31: n = 6 (NaCl), n = 6 (LiCl); cleaved Caspase 3: n = 6 (NaCl), n = 4 (LiCl); cleaved Caspase 3 cell pellet: n = 1 (/NaCl and LiCl)). Scale bar represent 200 μm (left) and 50 μm (right). (c) Immunofluorescence staining with anti-α-smooth muscle actin-Cy3 antibody was performed on paraffin-embedded sections (3 μM). Staining intensity was measured with ImageJ 1.47v software and normalized to tumor size. [file 12943_2015_294_MOESM6_ESM.pdf]

Suppl. Fig. 7

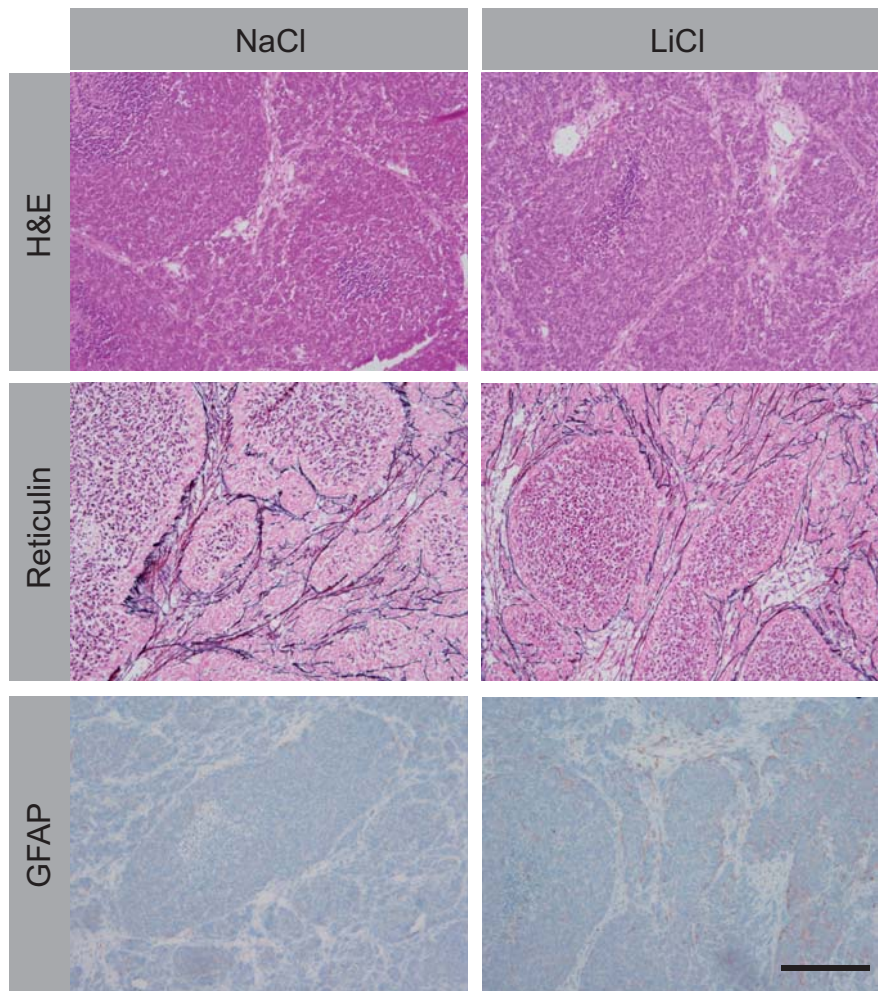

Supplement: Additional file 7: Figure S7. — Histo-pathological characterization of subcutaneous Ptch+/- tumors. Paraffin-embedded sections (3 μm) of flank tumors were stained with hematoxylin and eosin. Immunohistochemistry with anti-GFAP antibody (DakoCytomation) and reticulin (special stain Ventana) on paraffin-embedded sections (3 μm) of flank tumors was performed using the automated Ventana Discovery XT staining system (Ventana, Tucson, Arizona USA) and standard protocols. Stainings were visualized by Olympus BX50 light microscope 10× (NA 0.30). Scale bar represent 200 μm. [file 12943_2015_294_MOESM7_ESM.pdf]
